# Single-Cell Multiplex Immune Biomarker Prediction from Histopathology Across Cancer Types and Clinical Endpoints

**DOI:** 10.21203/rs.3.rs-11002687/v1

**Published:** 2026-10-05

**Authors:** Usman Afzaal, Usama Sajjad, Abdul Rehman Akbar, Tianyang Wang, Elshad Hasanov, Sachin Jhawar, Wei Chen, Zaibo Li, Muhammad Khalid Khan Niazi

**Affiliations:** 1 Department of Pathology, College of Medicine, The Ohio State University Wexner Medical Center, Columbus, OH, USA; 2 Department of Medical Oncology, College of Medicine, The Ohio State University Wexner Medical Center, Columbus, OH, USA; 3 Department of Radiation Oncology, College of Medicine, The Ohio State University Wexner Medical Center, Columbus, OH, USA

## Abstract

**Background:**

The spatial organization of cellular lineages within the tumor microenvironment governs disease progression and therapeutic response, but multiplexed spatial profiling remains costly, technically complex, and tissue-destructive. Routine hematoxylin and eosin (H&E) histopathology is inexpensive and widely available but lacks explicit molecular information. This study aims to determine whether single-cell, biomarker-informed cellular states can be inferred directly from H&E images to enable scalable, non-destructive spatial biology across cancer types and clinical endpoints.

**Methods:**

We present CellPlexer, a deep learning framework trained on more than 36 million co-registered cells from paired H&E and multiplex immunofluorescence images to jointly segment nuclei and classify each cell into one of 15 hierarchically defined biomarker states. A spatially aware transformer integrates single-cell embeddings with patch-level histomorphology through attention-based multimodal fusion, generating a multi-scale whole-slide representation for clinical prediction. We validated CellPlexer across four independent cohorts spanning lung, breast, and colorectal cancer.

**Results:**

Here we show that CellPlexer consistently outperformed morphology-only baselines across recurrence, treatment response, survival, and mutation prediction tasks, achieving an AUC of 72.19% for pathological complete response prediction in triple-negative breast cancer (75.06% with clinical variable integration) and 83.09% for BRAF mutation prediction in colorectal cancer.

**Conclusions:**

These results demonstrate that morphologically inferred, single-cell biomarker states capture clinically and biologically meaningful tumor microenvironment organization, providing a scalable, non-destructive framework for extracting proteomic-level insights from widely available clinical histopathology archives.

## Introduction

Beyond the mere presence of specific cell populations, their spatial arrangement within a tumor is increasingly recognized as a key determinant of disease progression and treatment response. Understanding these spatial architectures has therefore emerged as a definitive frontier in decoding complex tissue ecosystems [1-4]. The tumor immune microenvironment (TIME) represents a highly complex spatial ecosystem composed not only of malignant cells, but also diverse non-malignant populations including immune cells, cancer-associated fibroblasts, endothelial cells, and pericytes embedded within an altered extracellular matrix [5]. Understanding how these distinct cell populations are spatially arranged, and how their topological interactions regulate tumor growth, invasion, metastasis, and therapy response, remains a central challenge in modern oncology[2,5]. Recent advances in high-dimensional spatial profiling technologies have enabled the sub-cellular mapping of multiple protein biomarkers, revealing how localized cellular neighborhoods form functional niches associated with therapeutic resistance and response. In particular, multiplexed immunofluorescence (mIF) techniques such as Co-Detection by Indexing (CODEX) imaging enable simultaneous in situ detection of numerous targets, providing insights into tissue topography and cellular networks that are unattainable by conventional staining approaches alone [6-11]. This capability has been further extended by highly multiplexed cyclic platforms like PhenoCycler[12], which capture 20–100 markers within a single tissue section through iterative imaging cycles, albeit at the risk of potentially compromising tissue integrity over extended processing. Conversely, single-cycle architectures like the Orion scanner enable the simultaneous detection of up to 20 markers, preserving tissue quality while delivering highly multiplexed profiles[9].

Despite their transformative potential, the widespread clinical and translational adoption of these spatial assays remains severely constrained by high reagent costs, labor-intensive workflows, specialized instrumentation, and limited scalability[1,12-14]. In contrast, hematoxylin and eosin (H&E) histopathology remains the clinical gold standard for tissue diagnostics, routinely generated at low cost across virtually all cancer workflows and serving as a ubiquitous, high-resolution source of tissue architecture, nuclear morphology, and cellular organization [15]. However, while H&E readily reveals morphological information, it remains limited in its ability to directly capture the precise molecular and functional states increasingly required for modern precision medicine. While such complex sub-visual patterns may elude human observers[16], they can potentially be inferred through advanced artificial intelligence frameworks capable of learning latent relationships between tissue histomorphology and molecular phenotype[5,13]. Bridging routine H&E histology with multiplex spatial analysis therefore offers a compelling and practical pathway to unlock scalable spatial biology insights directly from standard pathology imaging.

Driven by the emerging premise that histomorphology encodes rich molecular and cellular information, recent computational efforts have begun to explore the automated prediction of multiplex protein expression directly from H&E images. Wu et al.[13] introduced a deep learning framework trained on more than 1,300 paired H&E and CODEX mIF samples spanning over 16 million cells, enabling the virtual multiplex staining of 50 biomarkers from routine histology. Similarly, HEX[14] utilized pathology foundation models to predict 40 biomarkers associated with immune, structural, and functional programs, further demonstrating the downstream utility of virtual spatial proteomics for clinical outcome prediction. At an even larger scale, GigaTIME leveraged paired H&E and multiplex imaging data comprising approximately 40 million cells to generate virtual multiplex immunofluorescence images across 21 protein channels[5]. Complementing these, approaches such as MIPHEI-ViT[17], have further explored pixel-level prediction of mIF signals from H&E across diverse biomarker panels spanning immune, stromal, epithelial, and proliferative programs.

Despite this rapid progress, existing approaches remain fundamentally limited in their ability to map virtual biomarker predictions onto biologically grounded, cell-level representations. Methods such as GigaTIME[5] and HEX[14] frame the problem as patch- or pixel-level image translation. While effective at generating aggregated marker intensity maps across tissue tiles, these models cannot resolve which individual cells express specific markers, nor can they disentangle the distinct contributions of co-resident tumor, immune, and stromal populations within a shared spatial neighborhood. Even HEX, which advances the field by linking tile-level virtual spatial proteomics to clinical outcomes via multimodal fusion, still conflates multiple phenotypically distinct cell populations within its fundamental prediction unit. Conversely, a separate class of methods, including MIPHEI-ViT[17], ROSIE[13], and Immunocto[18], achieves single-cell resolution by generating per-cell biomarker phenotype labels directly from H&E morphology. However, these approaches treat cellular phenotyping as a terminal output, failing to propagate these structured, single-cell representations into downstream patient-level outcome modeling. Consequently, across both methodologies, the rich discriminative features learned during biomarker inference are lost to downstream clinical models. Where biomarker inference and outcome prediction are connected, as in HEX, the linkage operates at the tile level rather than the single-cell level. While recent efforts have begun to bridge this gap by demonstrating the feasibility of using H&E-derived cellular embeddings to predict clinical outcomes, these approaches have been strictly limited to a small, coarse set of morphologically defined populations[19]. As a result, the intricate local ecosystems formed by interacting cells within tissue microenvironments remain largely unmodeled, limiting the capacity of current frameworks to capture the cellular heterogeneity and spatial topology that underlie disease progression and therapeutic response.

To address these limitations, we introduce CellPlexer, a unified deep learning framework that transforms standard H&E whole-slide images into spatially resolved, biomarker-informed cellular representations and leverages these representations for downstream clinical prediction. CellPlexer operates at cell-level resolution, inferring the biomarker state of individual cells directly from H&E morphology across a panel of 15 markers spanning tumor, immune, stromal, and functional programs, trained on a corpus of 36 million annotated cells. A transformer-based encoder generates highly expressive per-cell embeddings that capture both nuclear morphology and local tissue context [20]. Critically, these embeddings serve a dual purpose within the framework: they simultaneously support single-cell biomarker classification and, through progressive aggregation, encode tissue-level cellular composition and spatial organization for clinical outcome prediction. An attention-based multimodal fusion module integrates the resulting cell-level representations with patch-level histomorphological features, learning how cellular phenotype and tissue architecture jointly determine clinical phenotype. This end-to-end design yields a single, coherent representation of each slide that preserves both fine-grained cellular detail and global tissue context, without requiring any molecular assay beyond the routine H&E stain. We demonstrate that CellPlexer achieves consistent and generalizable improvements across multiple cancer cohorts and clinical endpoints, suggesting it learns a broadly transferable representation of the tumor microenvironment. In a nutshell, these results establish that conventional H&E slides encode latent spatial cellular information that can be approximated at single-cell resolution, positioning CellPlexer as a scalable and biologically interpretable complement to high-plex spatial profiling technologies and opening a new avenue for molecularly informed clinical decision-making from routine histopathology (Figure 1).

## Results

### Biomarker-informed cellular representations improve recurrence risk stratification in the internal ILA cohort

To evaluate whether CellPlexer-derived cellular representations capture clinically meaningful recurrence-associated tissue features, including patterns related to cellular proliferation, immune composition, macrophage states, and stromal and vascular organization, we assessed the framework on an independent internal Invasive Lung Adenocarcinoma (ILA) cohort comprising 456 whole-slide images. Under a five-fold patient-wise cross-validation framework, CellPlexer achieved higher overall performance across all evaluated methods, reaching an AUC of 68.89 ± 5.35% and an accuracy of 63.22 ± 4.66%, outperforming attention-based multiple instance learning (ABMIL), CLAM, and TransMIL. CellPlexer additionally achieved a specificity of 65.85 ± 5.42% while maintaining competitive sensitivity of 59.18 ± 6.33%. In comparison, ABMIL achieved an AUC of 66.57 ± 5.55%, CLAM reached 66.13 ± 5.90%, and TransMIL achieved 64.93 ± 6.25%. Beyond classification performance, CellPlexer also achieved a concordance index of 64.75 and produced clear separation between predicted low- and high-risk patients in Kaplan-Meier survival analysis, yielding a hazard ratio of 2.20. These results indicate that the model’s representations capture clinically relevant tumor microenvironment organization associated with recurrence risk, enabling improved patient stratification beyond conventional morphology-only slide representations (Figure 2, Supplementary Table S11).

### Integration of biomarker-aware pathology features and clinical variables enhances pCR prediction in TNBC

We next evaluated CellPlexer in a treatment response prediction setting using an internal TNBC cohort comprising 166 whole slide images (WSIs). Under a five-fold patient-wise cross-validation framework, CellPlexer achieved higher overall discriminative performance than all evaluated approaches, reaching an AUC of 72.19 ± 4.48%, exceeding ABMIL (68.65 ± 5.94%), CLAM (67.97 ± 4.97%), TransMIL (69.53 ± 5.12%), and Pathrosetta (64.77 ± 6.18%). The framework additionally achieved a numerically higher sensitivity (69.06 ± 4.35%), indicating improved identification of patients likely to achieve pCR following therapy. Overall classification accuracy reached 66.24 ± 1.79% for CellPlexer, remaining comparable to the strongest morphology-only baselines while providing substantially improved overall discrimination.

To further assess whether clinical metadata provides complementary information to CellPlexer-derived histopathological representations, we integrated slide-level embeddings with routinely available clinicopathologic variables, including Age, Pre-NAC grade, HER2 IHC, HER2 Ratio, HER2 Copy #, and tumor type. As shown in Figure 3, multimodal integration further improved predictive performance, demonstrating that biomarker-informed tissue representations and clinical variables capture complementary dimensions of treatment response biology.

We additionally investigated the relative contribution of individual biomarker phenotypes to pCR prediction by independently training models using embeddings derived from each biomarker class separately. Immune-associated populations, particularly CD45+ immune cells and cytotoxic T-cell–related phenotypes, demonstrated strong predictive value. Tumor epithelial populations marked by Pan-CK also contributed substantially, suggesting that both tumor-intrinsic morphology and immune microenvironment organization are important determinants of therapeutic response in TNBC (Figure 3, Supplementary Tables S10 and S13).

### CellPlexer generalizes across independent survival prediction cohorts

To evaluate the generalizability of CellPlexer across distinct tissue types, we assessed the framework on two independent survival prediction cohorts spanning breast and colorectal cancer. On the TCGA-BRCA cohort, CellPlexer achieved an AUC of 71.43 ± 5.09% and an accuracy of 67.84 ± 3.14%, outperforming ABMIL, CLAM, and TransMIL. CellPlexer additionally achieved a specificity of 72.47 ± 13.67% while maintaining a competitive sensitivity of 59.56 ± 22.43%. In comparison, ABMIL achieved an AUC of 66.04 ± 5.92%, CLAM reached 65.27 ± 6.01%, and TransMIL achieved 58.52 ± 5.88%. We next evaluated CellPlexer on the independent CRC-SurGen cohort for colorectal cancer survival prediction, where CellPlexer had an AUC of 68.28 ± 5.31% with 65.11 ± 4.68% accuracy while maintaining a balanced sensitivity-specificity profile (54.68 ± 7.13% sensitivity and 71.49 ± 3.71% specificity). Although TransMIL achieved markedly higher specificity (98.71 ± 2.42%), this occurred at the expense of substantially reduced sensitivity (5.51 ± 8.22%), indicating poor identification of high-risk patients. These findings demonstrate that CellPlexer’s representations generalize across multiple organ systems and capture prognostically relevant tumor microenvironment organization associated with patient outcome (Figure 4a, b, Supplementary Tables S9 and S12).

### CellPlexer's cell-level phenotype representations enable mutation prediction from routine H&E histology

To investigate whether CellPlexer's cell-level biomarker representations capture tissue features associated with clinically important genomic alterations, we assessed the framework's performance on KRAS and BRAF mutation prediction in the CRC-SurGen cohort. KRAS and BRAF mutations are known to reshape immune infiltration, stromal architecture, and angiogenesis within the tumor microenvironment [21-23], producing histomorphological signatures that prior deep learning studies have shown are recoverable from routine H&E images [16,24], making them a biologically plausible target for CellPlexer's cell-level representations. For BRAF mutation prediction, given the pronounced class imbalance in the training set (195 wild-type vs. 39 mutant cases), we additionally report balanced accuracy alongside standard metrics. For this, CellPlexer again achieved a numerically higher overall AUC (83.09 ± 6.59%) while also attaining higher sensitivity (75.76 ± 10.77%) and balanced accuracy (75.46 ± 5.36%), indicating substantially improved detection of mutation-positive patients. Although CLAM achieved a marginally higher overall accuracy (81.86 ± 2.80%) through very high specificity (89.28 ± 5.41%), this occurred at the expense of considerably lower sensitivity (46.36 ± 24.08%). In contrast, CellPlexer maintained a substantially more balanced predictive profile across positive and negative classes. These findings suggest that these H&E-derived representations capture biologically meaningful tissue architecture and tumor microenvironment organization associated with clinically relevant genomic alterations, despite the model being trained solely to predict single-cell biomarker phenotypes rather than molecular expression levels (Figure 4c, d, Supplementary Tables S14 and S15).

## Discussion

A central challenge in computational pathology is that routine H&E histology captures rich tissue morphology but lacks explicit molecular information. CellPlexer addresses this gap by combining nuclear segmentation[20] with biomarker-informed single-cell representation learning and downstream clinical modeling. Unlike virtual-staining approaches that terminate at biomarker prediction or morphology-only MIL methods that rely primarily on tissue-level features, CellPlexer preserves cell-level information and local spatial context for patient-level prediction. Training on an ORION-derived cohort comprising more than 36 million annotated cells[9] provides the scale needed to learn representations spanning diverse cellular and microenvironmental states.

A key differentiator of CellPlexer is its hierarchical approach to integrating representations across multiple biological scales, beginning with accurate single-cell phenotype assignment across the 15-biomarker panel (Figure 5a, b). Spatial-aware attention first summarizes local cellular ecosystems within each patch, capturing contextual interactions among neighboring cells. CellPlexer subsequently integrates these cell-derived representations with morphology-derived embeddings[25]. Finally, attention-based MIL aggregates patch-level information into a global slide representation for downstream prediction (Figure 6)s. Through this hierarchical design, CellPlexer effectively learns representations spanning nucleus-level morphology, local tissue topology, and whole-slide organization within a unified framework. Consequently, CellPlexer consistently performed better than the strongest morphology-only baseline across all four benchmark cohorts (Figures 2-4), spanning three cancer types and multiple clinical endpoints, without requiring any task-specific architectural modifications. The performance gain was greatest for TCGA-BRCA survival prediction and smallest for CRC-SurGen, while on the internal TNBC cohort the framework achieved the highest overall discriminative performance for pathological complete response prediction, with an AUC of 72.19 ± 4.48%. Integration of routinely available clinicopathologic variables further increased the AUC to 75.06%, indicating that clinical information provides complementary predictive value beyond the histopathology-derived cellular representations alone. These results suggest that the learned representations capture clinically relevant features that generalize across distinct organs, disease processes, and clinical endpoints rather than overfitting to a single tissue context.

These head-to-head comparisons are particularly notable given that the competing methods already represent strong and widely adopted MIL baselines. In TCGA-BRCA, the framework exceeded ABMIL[26], CLAM[27], and TransMIL[28] in AUC, in the lung and CRC recurrence cohorts, it maintained a substantially more balanced and stable sensitivity-specificity tradeoff than competing approaches, while in TNBC, it improved discrimination between responders and non-responders while remaining competitive in overall metrics. These observations suggest that explicitly modeling cellular representations contributes relevant information that is not fully captured by morphology-only representations.

Beyond aggregate benchmark performance, the granular analyses provide insight into the biological programs captured by CellPlexer. In the TNBC experiments, models trained independently on embeddings derived from specific biomarker phenotypes revealed that immune-associated populations, particularly CD45+ immune cells and cytotoxic T-cell–related states, were among the strongest predictors of pathological complete response, while Pan-CK–associated tumor epithelial populations also contributed substantially. These findings are biologically coherent, as therapeutic response in TNBC is known to depend on both tumor-intrinsic programs and the surrounding immune microenvironment[29]. The predictive value retained by distinct cell types suggests that CellPlexer captures biologically meaningful cellular programs beyond generic H&E texture. Its cell-level phenotype assignments and embeddings also enable interrogation of the cellular populations and spatial configurations associated with clinical outcomes, as further examined through MIL attention analysis. Across the TNBC cohort, proliferative and cytotoxic T-cell markers showed the greatest attention selectivity in slides achieving pathological complete response, while regulatory and memory T-cell markers were comparatively de-emphasized (Supplementary Figure S4, with representative single-slide examples shown in Supplementary Figures. S2 and S3). This localization was not uniform across tasks. Benchmarked against an equally sized random patch subset, attention gave a real advantage for TCGA-BRCA survival prediction, while for ILA recurrence, both the attended and random subsets collapsed to near-chance levels (Supplementary Figures S7 and S8), indicating that for this task, restricting inference to just 5% of patches is insufficient to support prediction regardless of how those patches are selected.

In addition, to assess whether these learned cellular phenotypes reflect genuine molecular biology rather than pattern-matching confined to the training distribution, we performed an independent, transcriptome-based validation using HEST-1k, an external multi-organ resource of paired spatial transcriptomics and H&E imaging never seen during training. Across four immunologically and vascularly relevant Hallmark pathways, CellPlexer-predicted biomarker-positive cells were significantly enriched in transcriptomically high-scoring spots relative to low-scoring spots within the same tissue section, most notably for Angiogenesis, where CD31+ and SMA+ cells showed pathway-restricted enrichment (2.2- and 2.6-fold, FDR-corrected P < 0.001) (Supplementary Figs. S9–S12). This independent, transcriptome-derived concordance, observed on tissue types and platforms absent from training, indicates that CellPlexer's cell-level phenotype calls capture spatially organized biology rather than artifacts of the ORION training distribution.

Despite these strengths, several limitations warrant consideration. First, CellPlexer is computationally heavier than simpler morphology-only MIL frameworks such as ABMIL. This increased complexity arises from the need to perform spatial-aware attention, multimodal fusion, and slide-level aggregation. Consequently, the framework requires much longer training times and larger memory footprints relative to simpler baselines. In addition, although CellPlexer consistently improved overall performance across the evaluated cohorts, the magnitude of improvement over strong baselines was modest for some clinical endpoints. This suggests that the current strategy for integrating cellular representations with tissue-level morphology may not yet fully exploit the available biological information. Future work should therefore explore alternative aggregation and fusion strategies, including graph-based, hierarchy-aware, or task-specific approaches, to better leverage cell-level representations and potentially achieve larger gains in clinical outcome prediction.

Finally, one of the most important aspects of CellPlexer is that it does not collapse the learned biology into a single opaque prediction score. Instead, the framework produces reusable transformer-derived cell embeddings together with biologically interpretable cell-type assignments. In this sense, CellPlexer functions not only as a predictive model, but as a general representation-learning framework for the tumor microenvironment. Because these embeddings preserve cellular identity, morphology, and local spatial context, future work can explore alternative strategies for downstream integration beyond the current low-rank fusion and attention-MIL paradigm, including graph-based aggregation, hierarchy-aware pooling, spatial foundation models, or task-specific multimodal fusion approaches. This design also creates a broader opportunity for the computational pathology community. Different downstream clinical tasks may benefit from distinct methods for combining cell embeddings, biomarker phenotypes, and tissue morphology, and CellPlexer provides the intermediate cellular representations necessary to systematically investigate these possibilities. Accordingly, the primary contribution of this work may extend beyond the immediate benchmark improvements reported here. More fundamentally, CellPlexer introduces a biologically grounded and spatially resolved representation layer that enables future methodological development in computational spatial pathology and offers a scalable route toward molecularly informed clinical modeling directly from routine H&E histology.

## Conclusion

In conclusion, CellPlexer introduces a robust, biologically grounded framework for computational pathology by demonstrating that routine, low-cost H&E histopathology slides encode latent spatial cellular networks that can be accurately resolved at single-cell resolution. Rather than relying on ungrounded patch embeddings or noisy virtual-staining pixel translations, our architecture establishes a framework that treats individual cells as the foundational unit of tissue-level and clinical modeling. Independent validation across multiple cancer cohorts confirms that the integration of biomarker-informed cellular representations with multi-scale tissue-level morphology systematically performs better than standard morphology-only pipelines. Furthermore, mechanistic validation of predictive drivers yields biologically coherent cellular configurations that provide an essential layer of transparency and interpretability over traditional black-box histopathology slide models. Although the requirement for concurrent spatial-aware attention and multi-biological scale fusion imposes a heavier computational and preprocessing burden than simpler bag-of-patches approaches, the resulting reusable, high-dimensional cell embeddings lay a scalable foundation for future spatial tissue foundation models. Ultimately, CellPlexer bridges the critical gap between resource-intensive high-plex multiplex profiling and routine clinical diagnostics, unlocking a scalable and molecularly informed pathway for data-driven precision oncology.

## Methods

### Data and Preprocessing

#### Datasets

CellPlexer was trained and internally validated on the ORION dataset, which comprises co-registered H&E and multiplex immunofluorescence (mIF) whole-slide images (WSIs) from colorectal cancer patients acquired using the Orion cyclic imaging platform[9]. The ORION dataset provides simultaneously acquired H&E and 18-channel mIF images from the same tissue section, enabling pixel-level alignment between morphology and protein expression. The H&E images were acquired with an Aperio GT450 scanner (Leica Biosystems) at 0.325 microns per pixel (mpp), and co-registered to the corresponding mIF images [9]. The simultaneous co-staining protocol eliminates tissue deformation artifacts associated with consecutive-section designs, enabling direct pixel-level correspondence between morphology and protein expression, a prerequisite for training a cell-level biomarker prediction model. Of the 18 available mIF channels, we retained 15 markers for cell phenotyping. Following preprocessing and quality control, the dataset comprised 969,890 image patches partitioned at the patient level into training (660,409 tiles), validation (138,031 tiles), and test (171,450 tiles) sets. Cell-level annotations were derived from the mIF channels via automated marker thresholding, hierarchical gating, and spatial reconciliation with CellViT-derived nuclear segmentation polygons, yielding a total of 36,401,610 annotated cells across the 15 phenotype classes. The distribution of cell types across splits and the per-class cell counts are summarized in Figure 1 and Supplementary Table S1.

#### Image Preprocessing

Registration between H&E and mIF whole-slide images was performed as part of the original ORION data release[9], and we utilized the pre-registered image pairs directly for this study. Autofluorescence subtraction and channel normalization followed the pipeline described in MIPHEI [17], to which we refer the reader for complete details; we summarize the key steps here for completeness. Autofluorescence (AF), captured as an independent antibody-free control channel $I_{AF}$, was subtracted from each marker channel $I_{AF}^{c}$ using per-channel scaling and offset parameters $\lambda^{c}$ and $b^{c}$ manually adjusted using a Napari-based visualization interface [30] as shown in equation (1):

(1) $$I_{\text{cor}}^{c}=\max(0,I_{\text{IF}}^{c}-\lambda^{c}.\;I_{\text{AF}}+b^{c})$$

Each corrected channel was then normalized using the 99.9th percentile intensity $q_{0.999}^{c}$, computed from foreground pixels across the training set, and log-transformed to compress high-intensity values and reduce the influence of extreme outliers as in equation (2):

(2) $$I_{\text{norm}}^{c}=255.\log\left(\frac{\text{min}(I_{\text{cor}}^{c},q_{0.999}^{c})}{q_{0.999}^{c}}+1\right)$$

This normalization ensures a consistent dynamic range across markers and training samples.

### Cell Biomarker Panel and Hierarchical Class Assignment

To train the model, a 15-marker cell phenotyping panel was designed to comprehensively characterize the major cellular constituents and functional states of the tumor microenvironment. The panel encompassed epithelial, immune, stromal, vascular, proliferative, and immune-checkpoint programs, including Pan-CK (epithelial cells), CD45 (pan-immune), CD3e (pan-T cells), CD4 (helper T cells), CD8a (cytotoxic T cells), FOXP3 (regulatory T cells), CD20 (B cells), CD68 (macrophages), CD163 (macrophage-associated cells), CD45RO (memory-associated immune cells), PD-1 (immune checkpoint receptor), PD-L1 (immune checkpoint ligand), Ki-67 (cellular proliferation), CD31 (endothelial cells), and SMA (smooth muscle cells and myofibroblasts). Pan-CK and E-cadherin positivity were used to identify epithelial cells, because these markers can also be expressed by benign epithelium, epithelial marker positivity alone was not interpreted as evidence of malignant tumor identity. Together, these markers capture key cellular populations and immunoregulatory states associated with tumor progression, immune response, and therapeutic outcome. This panel was designed to capture not only lineage identity but also functional immune states, including checkpoint expression and proliferative activity, that are increasingly recognized as determinants of tumor behavior and response to immunotherapy[31]. Per-cell marker positivity was determined by thresholding each autofluorescence-corrected and normalized mIF channel, generating a binary marker expression profile across all 15 biomarkers for every annotated cell. To convert these multi-marker positivity vectors into biologically interpretable cell phenotypes, a deterministic hierarchical gating strategy was applied that enforces mutual exclusivity and follows established immunophenotyping principles. The hierarchy first assigns tumor epithelial identity, followed by progressive immune lineage and subtype classification, including macrophage polarization states, B-cell populations, and increasingly specialized T-cell subsets, before finally resolving stromal and structural cell populations. Each cell was assigned exactly one label, ensuring a mutually exclusive and exhaustive partition across all 15 classes, with no cell left unclassified. The complete gating logic and priority ordering are detailed in Supplementary Figure S1.

### Cell Segmentation, Quality Control, and Ground Truth Construction

Individual cell nuclei were segmented from H&E whole-slide images using CellViT[20], a vision transformer–based framework for panoptic nuclear segmentation. Applied at 40× magnification, CellViT generated high-resolution nuclear boundary polygons for individual cells across the tissue section. In parallel, cell centroid coordinates and corresponding phenotypic labels were independently derived from the multiplex immunofluorescence (mIF) data following the preprocessing strategy described in MIPHEI[17]. Specifically, cell identities were assigned through hierarchical gating of thresholded biomarker channels, producing a spatially resolved set of marker-defined cellular phenotypes, as defined before.

Because the H&E-derived nuclear segmentations and mIF-derived cellular labels originate from independent imaging and processing pipelines, they are not intrinsically aligned at the single-cell level. The mIF pipeline provides centroid-level cellular annotations with associated biomarker-defined phenotypes, whereas CellViT produces morphology-driven nuclear polygons extracted directly from H&E morphology. Establishing paired training samples therefore required explicit spatial reconciliation between these complementary representations to associate each segmented nucleus with its corresponding molecular phenotype.

To associate biomarker-defined cellular phenotypes with H&E-derived nuclear segmentations, a multi-stage spatial reconciliation procedure was developed to map each CellViT polygon to a corresponding mIF-derived cell label. In the first stage, each mIF-derived centroid was evaluated for spatial containment within the segmented nuclear polygons using point-in-polygon analysis. Polygons containing a single centroid were assigned the corresponding cellular phenotype, whereas polygons containing multiple centroids with consistent labels retained that shared class assignment. Cases containing conflicting phenotypic labels within the same polygon were considered ambiguous and excluded to preserve label fidelity.

In the second stage, mIF centroids that were not spatially enclosed by any segmented nucleus, typically arising from minor registration offsets or imperfect nuclear boundary delineation, were matched to the nearest unassigned polygon within a constrained spatial threshold. When no suitable polygon could be reliably associated, a synthetic nucleus approximation centered at the mIF coordinate was generated to preserve biologically valid marker-positive cells that would otherwise be lost due to minor segmentation or alignment discrepancies. This strategy enabled retention of informative cellular phenotypes while avoiding corruption of confidently matched nuclei.

Finally, remaining unlabeled CellViT polygons were matched to nearby mIF centroids based on spatial proximity. Nuclei without reliable phenotypic association after reconciliation were discarded from downstream training. The resulting dataset consisted of high-confidence nuclear polygons paired with biologically resolved single-cell phenotype labels spanning 15 cellular classes and was subsequently used to train CellPlexer for large-scale cell phenotype classification. Model training was performed on 24 NVIDIA A100 GPUs.

### Whole-Slide Image Feature Extraction

Each WSI was divided into non-overlapping patches of 256 × 256 pixels at 20× magnification. Patch-level embeddings of dimension $d_{p}=1536$ were extracted using UNIv2 [25], a large pathology vision foundation model pretrained on diverse histopathological images, with weights held fixed throughout CellPlexer training. For patches containing detected cells, a 1,280-dimensional embedding was extracted for each cell from the penultimate layer of the fine-tuned CellViT encoder, capturing the nuclear morphology of the individual cell. Each cell was associated with the patch in which its centroid resided. To ensure complete and unbiased coverage of the entire tissue section, both patches containing detected cells and patches with no detected cells were retained and processed, where patches without cells contribute patch-level morphological features to the slide representation without a cell-level component, ensuring that stromal expanses, necrotic regions, and other acellular tissue compartments are not excluded from the model's input.

### CellPlexer Architecture

CellPlexer is a deep learning framework that transforms a standard H&E whole-slide image into a spatially resolved, biomarker-informed cellular representation and leverages it for clinical outcome prediction. The framework operates through three successive stages: (1) cell-level aggregation within each patch via spatial-aware attention, producing a single patch representation informed by the identity, morphology, and spatial arrangement of individual cells; (2) fusion of this cell-derived representation with a morphology-derived patch embedding via low-rank tensor fusion [32], integrating complementary cellular and tissue-level signals into a unified per-patch feature; and (3) slide-level aggregation of all patch representations via attention-based multiple instance learning, which produces a single slide embedding used for downstream clinical outcome prediction (Figure 6).

### Spatial-Aware Attention for Cell-Level Aggregation

For each patch containing N detected cells, let $e_{i}\in R^{d_{c}}$ denote the CellPlexer embedding of the ith cell and $c_{i}\in R^{2}$ its centroid coordinates normalized within the local patch. To preserve spatial context, positional information is encoded using sinusoidal positional embeddings [33] and added to the cell embeddings, producing spatially aware cellular representations as in equation (3):

(3) $$\tilde{e_{ı}}=e_{i}+\emptyset (c_{i})$$

where ∅(⋅) denotes the positional encoding function. These enriched embeddings jointly encode cellular morphology and spatial organization within the local tissue microenvironment. A learnable CLS token $e_{CLS}\in R^{d_{c}}$ is then prepended to the cell sequence, forming the input $X=[e_{CLS};\;\tilde{e_{ı}};\ldots ;\tilde{e_{N}}]\in R^{(N+1)\times d_{c}}$. Equation (4) shows self-attention, which is applied across the full cellular sequence:

(4) $$Z=\text{softmax}\left(\frac{{\mathrm{QK}}^{T}}{\sqrt{d_{h}}}\right)V\in R^{(N+1)\times d_{h}}$$

where Q,K, and V are linear projections of X, and $d_{h}$ denotes the attention dimensionality. Through this operation, the model learns contextual interactions between neighboring cells and selectively aggregates biologically relevant cellular states across the patch. The output corresponding to the CLS token serves as a compact cell-level representation $h_{cell}\in R^{d_{h}}$, summarizing the phenotypic composition and spatial arrangement of the local cellular ecosystem.

### Low-Rank Tensor Fusion for Patch-Level Fusion

While the cell-derived representation captures local cellular identity and spatial organization, it does not fully represent broader tissue architecture and histomorphological context. To incorporate complementary morphological information, tissue-level UNIv2 patch embeddings $p\in R^{d_{p}}$ were projected into the same latent space, denoted as $p^{\prime }$. The cellular and tissue-level modalities are subsequently integrated using low-rank tensor fusion. Bias-augmented representations are first constructed:

$$\hat{h}=[h_{\text{cell}};\;1],\;\;\hat{p}=[p^{\prime };\;1]$$

Fusion is then performed through low-rank factorized interactions as in equation (5):

(5) $$f=(W_{1}\hat{h})\;⊙\;(W_{2}\hat{p})$$

where ⊙ denotes element-wise multiplication. The final fused patch representation is obtained using equation (6):

(6) $$h_{patch}=W_{3}f$$

Unlike simple concatenation, this formulation efficiently captures higher-order multiplicative relationships between cellular phenotypes and tissue morphology while remaining computationally tractable. The resulting representation jointly models cellular composition, tissue architecture, stromal organization, and broader histological structure within a unified latent space. Patches lacking cells bypass the cellular aggregation branch and are represented using tissue morphology alone, via a separate linear projection. All per-patch representations are then passed through a shared adapter layer that maps both pathways into a common representation space, ensuring compatibility during slide-level aggregation.

### Attention-Based MIL for Slide-Level Aggregation

Given a whole-slide image containing M patches with fused representations ${\left\{h_{\text{patch}}^{\left(m\right)}\right\}}_{m=1}^{M}$, CellPlexer employs gated attention-based multiple instance learning (MIL) to aggregate patch-level information into a global slide representation. Attention weights are computed using equation (7):

(7) $$a_{m}=W_{\text{att}}(\tanh({\text{Vh}}_{m})\;⊙\;\sigma ({\text{Uh}}_{m})),$$

where Vand U are learned projection matrices, σ(⋅) denotes the sigmoid activation function, and $W_{\text{att}}$ produces patch-specific attention scores. The gated attention mechanism enables the model to selectively prioritize clinically informative tissue regions while suppressing ambiguous or uninformative patches. Subsequently, normalized attention weights ${\hat{a}}_{m}$ are obtained using softmax normalization across all patches, and the final slide representation is computed using equation (8):

(8) $$s=\sum_{m=1}^{M}{\hat{a}}_{m}\;h_{m}.$$

The aggregated slide embedding s is then passed through a classification head to generate the final patient-level prediction. Through this hierarchical aggregation framework, CellPlexer integrates information across multiple biological scales, ranging from individual cellular phenotypes and local spatial neighborhoods to global tissue architecture and whole-slide disease patterns. Furthermore, to interpret which tissue regions and biomarker phenotypes contribute most to slide-level predictions, we subsequently analyzed the distribution of learned attention weights across biomarkers and tumor microenvironment (TME) compartments, and evaluated model performance using only the most-attended patches (Supplementary Figs. S2–S7).

### Training and Evaluation

CellPlexer was trained using cross-entropy, with class imbalance addressed through a positive class weight derived from the training fold label distribution. Parameters were optimized using Adam[34] with a learning rate of 10^−4^ and $\ell_{2}$ weight decay of 10^−5^. During training, multiple patches and their associated cells are processed together in a single batch, enabling efficient GPU utilization across the heterogeneous patch-cell structure of each WSI. Model performance was evaluated using five-fold cross-validation with patient-level stratified splitting, ensuring no patient overlaps across training, validation, and test sets within each fold.

To benchmark CellPlexer against established computational pathology methods, we compared performance against several widely used morphology-only multiple instance learning (MIL) frameworks, including attention-based MIL (ABMIL)[26], CLAM[27], and TransMIL[28]. These methods operate exclusively on patch-level histomorphological embeddings without incorporating explicit cell-level biomarker or spatial phenotype information. In addition, for the TNBC pathological complete response (pCR) prediction task, we additionally compared against PathRosetta, a recently proposed framework that integrates limited cell-level information through a small set of coarse cellular categories. Unlike CellPlexer, which models 15 biomarker-informed cellular phenotypes together with spatially contextualized single-cell embeddings, PathRosetta incorporates only five broad cell-type groupings for downstream prediction. H&E patches from these external cohorts were stain-normalized to the ORION reference distribution at inference time using the Vahadane method[35], with the reference stain matrix derived from a representative ORION patch.

As an additional baseline for the TNBC pCR and ILA recurrence tasks, we used GigaTIME, a pretrained model that predicts 23 virtual protein marker channels from H&E images, to generate a 21-dimensional multi-protein activation-density feature vector per slide, following the activation-density formulation described in the original GigaTIME publication[5]. Each channel's density is the proportion of GigaTIME-predicted positive pixels relative to total slide pixels. A logistic regression classifier was trained on these vectors under the same five-fold, patient-level cross-validation scheme as CellPlexer, with hyperparameters and the classification threshold selected via grid search on the validation set.

For colorectal cancer survival prediction, CellPlexer was evaluated on the public CRC-SurGen cohort, comprising a total of 424 WSIs. This cohort was used to assess the ability of the framework to capture prognostically relevant spatial and cellular patterns associated with survival in colorectal cancer. Patient-level dataset splits and detailed cohort statistics are provided in Supplementary Table S3.

For KRAS and BRAF mutation prediction, we used the same CRC-SurGen cohort, restricted to patients with available mutation status, and framed each task as binary classification. These tasks tested whether CellPlexer's cellular representations, learned without any molecular supervision, capture histologic correlates of clinically important genomic alterations. Cohort statistics and label distributions for both tasks are provided in Supplementary Tables S7 and S8.

For breast cancer survival prediction, we evaluated CellPlexer on the TCGA-BRCA cohort, consisting of 146 WSIs. This dataset enabled assessment of the framework’s ability to learn survival-associated histomorphological and cellular representations from routine H&E pathology images. Detailed dataset composition and split statistics are summarized in Supplementary Table S4.

For pathological complete response (pCR) prediction following neoadjuvant chemotherapy, we utilized an internal triple-negative breast cancer (TNBC) cohort containing 166 WSIs. This cohort was used to evaluate the framework in a treatment response prediction setting, where spatial interactions between tumor and immune populations play a critical role in therapeutic outcome. Detailed patient-level splits and clinical characteristics are provided in Supplementary Table S5.

For ILA recurrence prediction, CellPlexer was evaluated on an internal ILA cohort comprising 456 WSIs. This cohort enabled assessment of the generalizability of the framework across a distinct tissue type and clinical endpoint, further testing the robustness of the learned biomarker-informed representations for recurrence risk stratification. Detailed cohort composition and dataset splits are provided in Supplementary Table S6.

For all cohorts, evaluation was performed using five-fold cross-validation. Model performance was assessed using the area under the receiver operating characteristic curve (AUC), sensitivity, specificity, and accuracy, with results reported as mean ± standard deviation across the five cross-validation folds.

### Independent Validation using Spatial Transcriptomics

To determine whether CellPlexer's cell-level biomarker phenotypes correspond to genuine molecular biology beyond the internal ORION-derived test set, we performed an independent validation using HEST-1k[36], a multi-organ resource containing paired spatial transcriptomics and H&E whole-slide images. CellPlexer was applied directly to HEST slides without additional training or fine-tuning. Predicted cell centroids were assigned to their nearest spatial transcriptomics spot within a platformspecific radius set by spot diameter and pixel resolution. Spot-level gene expression was normalized and summarized into 50 Hallmark pathway scores[37]; for four immunologically and vascularly relevant pathways (Interferon Gamma Response, IL-2/STAT5 Signaling, Inflammatory Response, Angiogenesis), spots were stratified into high- and low-scoring quartiles per section. A biomarker was considered present if ≥3 CellPlexer-positive cells of that phenotype fell within a spot's assignment radius; enrichment in high- versus low-scoring spots was tested per biomarker–pathway pair with a one-sided Fisher's exact test, applying Benjamini–Hochberg FDR correction across all comparisons.

## Supplementary Material

This is a list of supplementary files associated with this preprint. Click to download.

• manuscriptcellplexerNCMSI.pdf

## Figures and Tables

**Figure 1:**
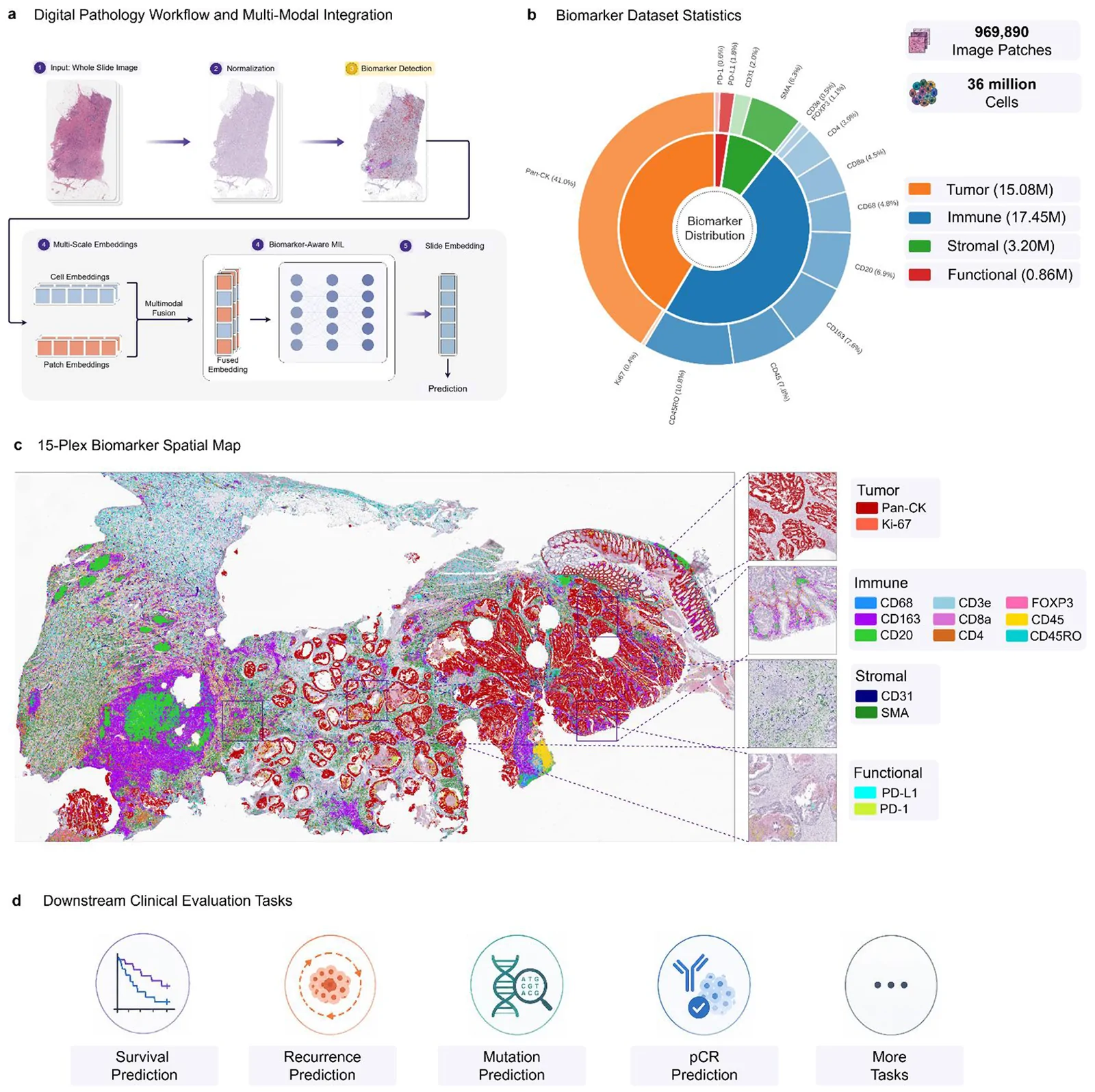
CellPlexer framework for large-scale single-cell biomarker inference and multimodal tissue representation learning from routine H&E histopathology. **a, End-to-end digital pathology workflow.** Whole-slide H&E images undergo stain normalization, CellViT-based nuclear segmentation [20], and single-cell biomarker prediction across 15 phenotypes. Cell-level embeddings are integrated with patch-level morphology features through a multimodal multiple instance learning (MIL) framework to generate slide-level clinical predictions. **b, Summary of the ORION-based biomarker training cohort used for CellPlexer development.** The dataset comprises 969,890 image patches and more than 36 million annotated cells derived from co-registered H&E and multiplex immunofluorescence (mIF) images. The plot illustrates the distribution of the 15 biomarker phenotypes across the dataset, including tumor epithelial (Pan-CK), immune (CD45, CD3e, CD4, CD8a, FOXP3, CD20, CD45RO), macrophage and checkpoint-related (CD68, CD163, PD-1, PD-L1), proliferative (Ki67), and stromal/vascular (SMA, CD31) populations. The large-scale cellular diversity enables robust learning of spatially resolved tumor microenvironment representations. **c, Spatially resolved biomarker mapping from H&E morphology.** Representative ORION whole-slide image demonstrating CellPlexer-derived spatial localization of 15 biomarker phenotypes across the tumor microenvironment. Insets highlight distinct tumor, immune, stromal, and functional niches alongside representative examples of tissue morphology, CellPlexer nuclear segmentation, and final biomarker assignments. **d, Downstream clinical applications of CellPlexer.** Illustration of the broad applicability of the CellPlexer framework across diverse computational pathology tasks, including survival prediction, recurrence prediction, mutation prediction, pathological complete response (pCR) prediction, molecular subtyping, and other biomarker-informed clinical outcome prediction settings.

**Figure 2:**
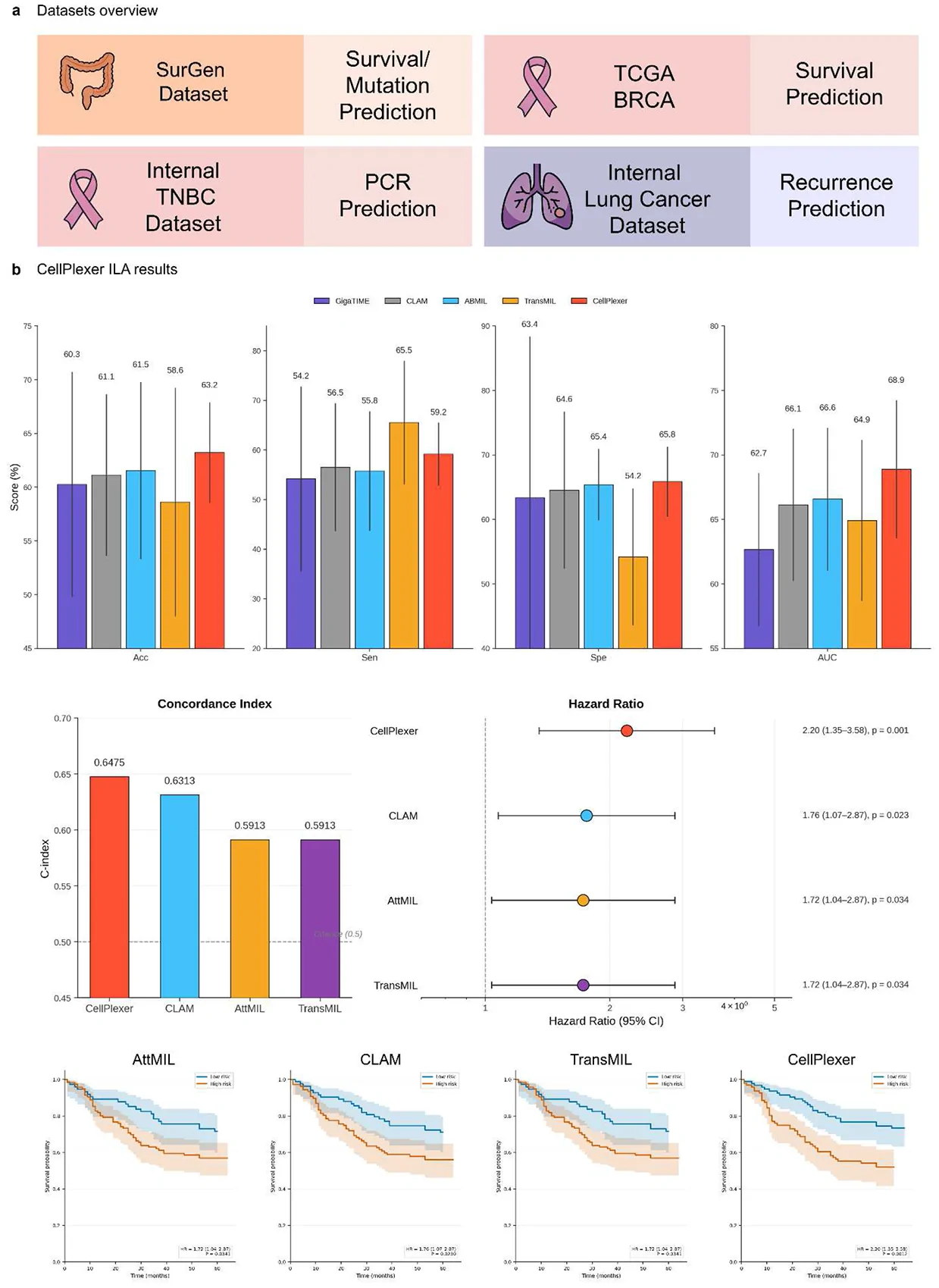
Multi-cohort benchmarking and generalization performance of CellPlexer across diverse cancer types and multiple clinical endpoints. **a, Independent evaluation cohorts and associated clinical prediction tasks.** Overview of the four independent evaluation cohorts spanning multiple organ systems and clinically relevant endpoints. Cohorts include TCGA-BRCA for breast cancer survival prediction, the public CRC-SurGen cohort for colorectal cancer survival prediction and gene mutation prediction, an internal triple-negative breast cancer (TNBC) cohort for pathological complete response (pCR) prediction following neoadjuvant chemotherapy, and an internal ILA cohort for recurrence prediction. Cohort sizes, tissue origins, and associated downstream tasks are shown for each cohort. **b, Recurrence prediction and survival stratification in the internal ILA cohort.** Comparative evaluation of CellPlexer against CLAM, ABMIL, and TransMIL using classification metrics including Accuracy (Acc), Sensitivity (Sen), Specificity (Spe), Area Under the ROC Curve (AUC), and Concordance Index (C-index). Kaplan-Meier survival curves for predicted low- and high-risk patient groups, with corresponding hazard ratios.Integration of biomarker-aware pathology features and clinical variables enhances pCR prediction in TNBC

**Figure 3:**
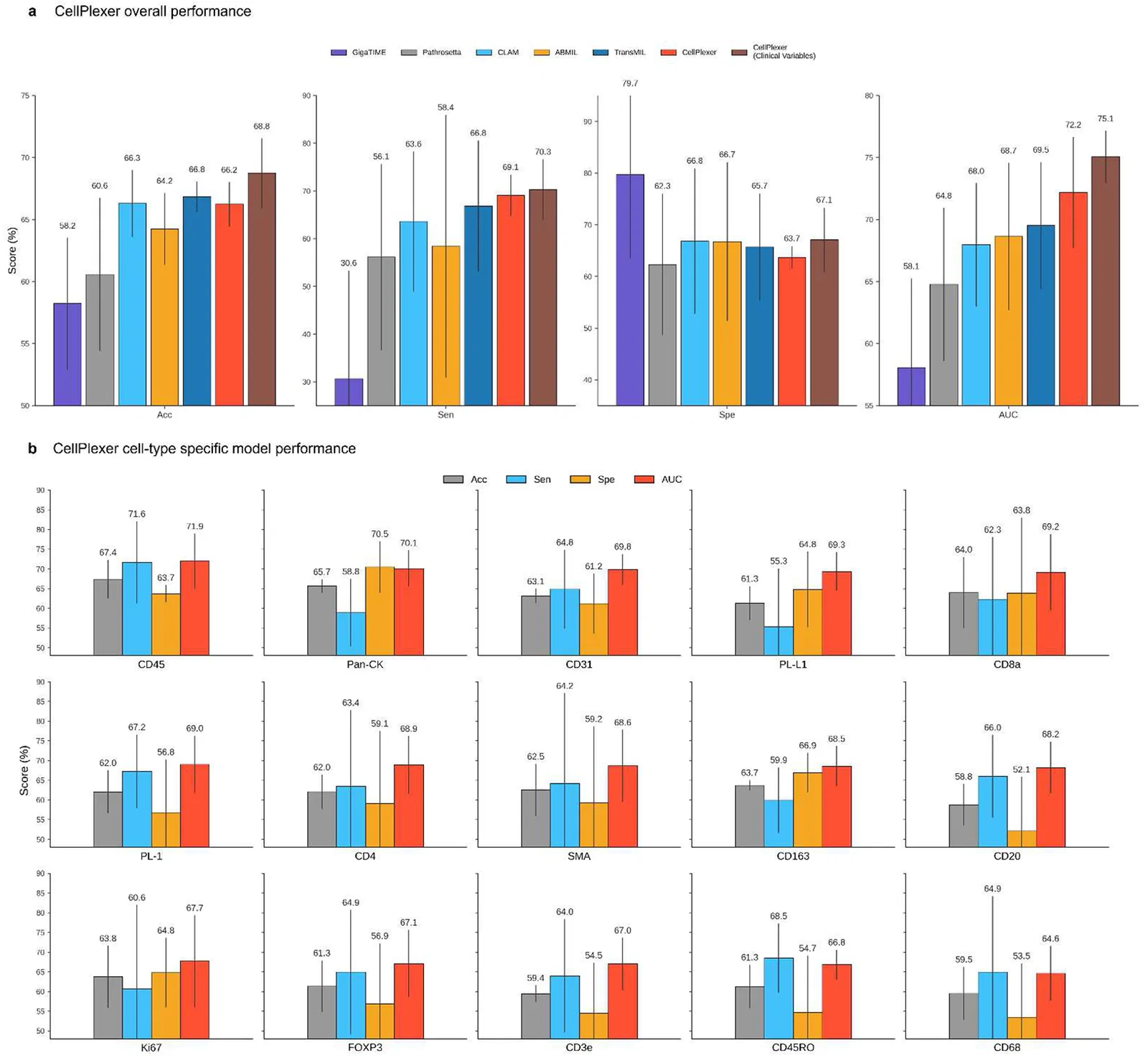
Pathological complete response prediction and biomarker-specific predictive drivers in triple-negative breast cancer. **a, Benchmarking of pCR prediction performance in the internal TNBC cohort.** Comparative evaluation of CellPlexer against multiple state-of-the-art computational pathology baselines for pathological complete response (pCR) prediction following neoadjuvant chemotherapy. Metrics include classification accuracy and area under the receiver operating characteristic curve (AUC). Performance is also shown for the image-only CellPlexer model integrated with clinical variables. **b, Biomarker-specific contribution analysis for pCR prediction.** Predictive performance of CellPlexer when trained independently using embeddings derived from each of the 15 biomarker phenotype classes separately, including immune-associated populations (e.g., CD45+ immune cells, cytotoxic T-cell–related phenotypes) and tumor epithelial markers (e.g., Pan-CK).

**Figure 4:**
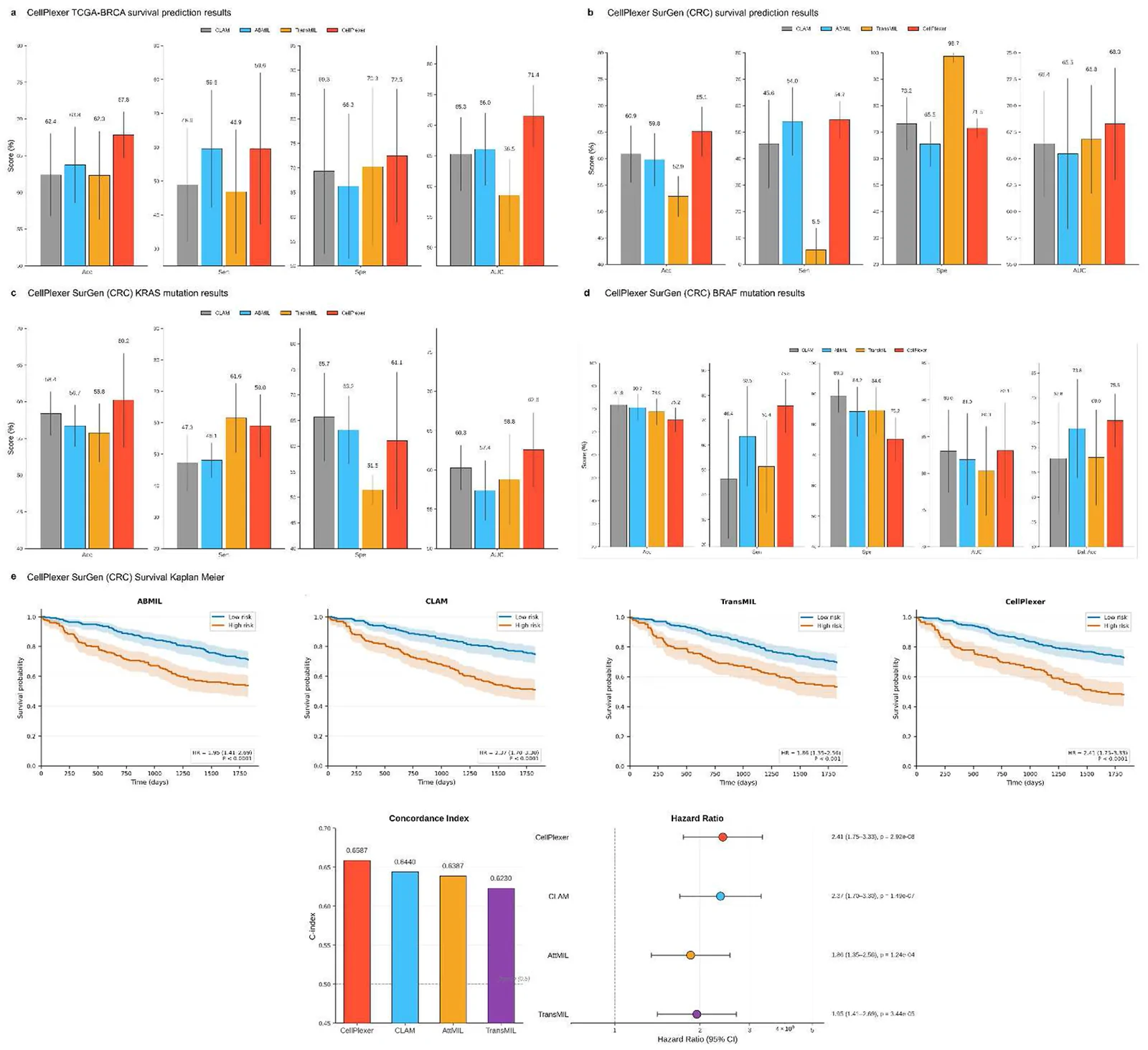
Generalization of CellPlexer across independent survival and mutation prediction tasks. **a, Breast cancer survival prediction on the TCGA-BRCA cohort.** Comparative evaluation of CellPlexer against established computational pathology baselines (CLAM, ABMIL, and TransMIL) using Accuracy (Acc), Sensitivity (Sen), Specificity (Spe), and Area Under the ROC Curve (AUC). **b, Colorectal cancer survival prediction on the public CRC-SurGen cohort.** Comparative evaluation of CellPlexer against ABMIL, CLAM, and TransMIL using the same classification metrics. **c, KRAS mutation prediction on the CRC-SurGen cohort.** Comparative evaluation of CellPlexer against baseline methods using the same classification metrics. **d, BRAF mutation prediction on the CRC-SurGen cohort.** Comparative evaluation of CellPlexer against baseline methods using Accuracy, Sensitivity, Specificity, AUC, and Balanced Accuracy, given the pronounced class imbalance in the training set.

**Figure 5:**
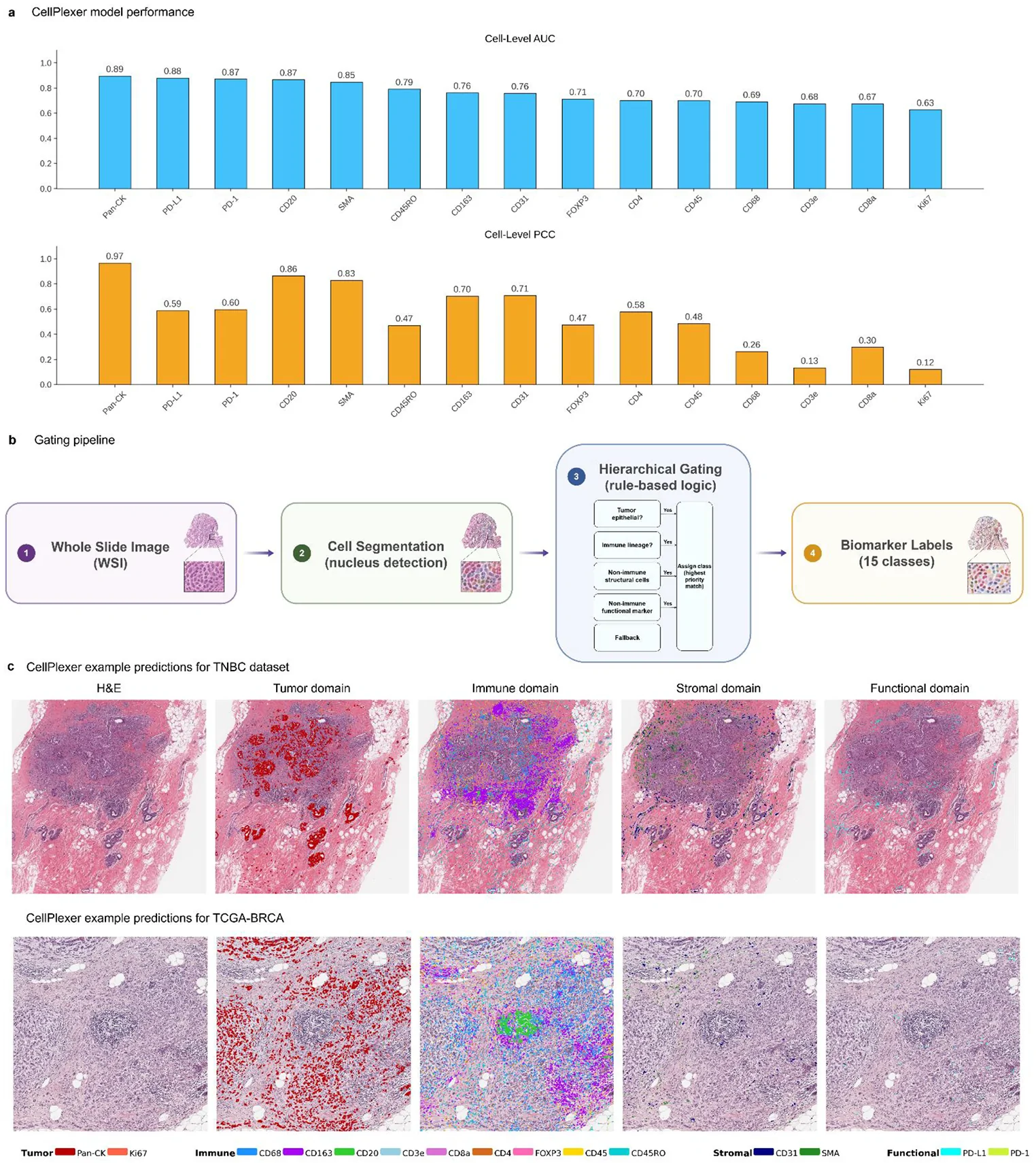
Large-scale single-cell biomarker learning, hierarchical phenotype assignment, and representation structure in CellPlexer. **a, Single-cell biomarker classification performance across the 15-plex panel.** Quantitative evaluation of CellPlexer biomarker inference across the 15 biomarker classes using Pearson correlation coefficient (PCC) and AUC. PCC measures the agreement between CellPlexer predictions and the reference biomarker annotations for each biomarker class, to visualize the recovery of biologically meaningful cellular phenotypes across epithelial, immune, stromal, proliferative, and immune checkpoint populations from routine H&E morphology. **b, Hierarchical gating and ground-truth generation pipeline for biomarker annotation.** Schematic representation of the workflow used to generate high-confidence cell-level biomarker labels from multiplex immunofluorescence data. Whole-slide images first undergo CellViT-based nuclear segmentation followed by marker thresholding and hierarchical immunophenotypic gating. The hierarchical assignment procedure prioritizes tumor epithelial identity before progressively resolving immune lineage and specialized cellular subtypes, enabling mutually exclusive assignment of each cell into one of the 15 biomarker phenotypes. Spatial reconciliation between mIF-derived labels and H&E-derived nuclei produces the final single-cell annotations used for CellPlexer training (see Supplementary Figure S1). **c, Representative CellPlexer biomarker prediction examples across breast cancer cohorts.** Visualization of CellPlexer-derived biomarker predictions in representative TNBC and TCGA-BRCA whole-slide regions. Predicted cellular phenotypes demonstrate spatial organization and biologically coherent clustering patterns within tumor, stromal, immune, and functional compartments. These examples illustrate the framework’s ability to generalize across distinct cancer cohorts while preserving fine-grained spatial heterogeneity and phenotypic structure at single-cell resolution.

**Figure 6:**
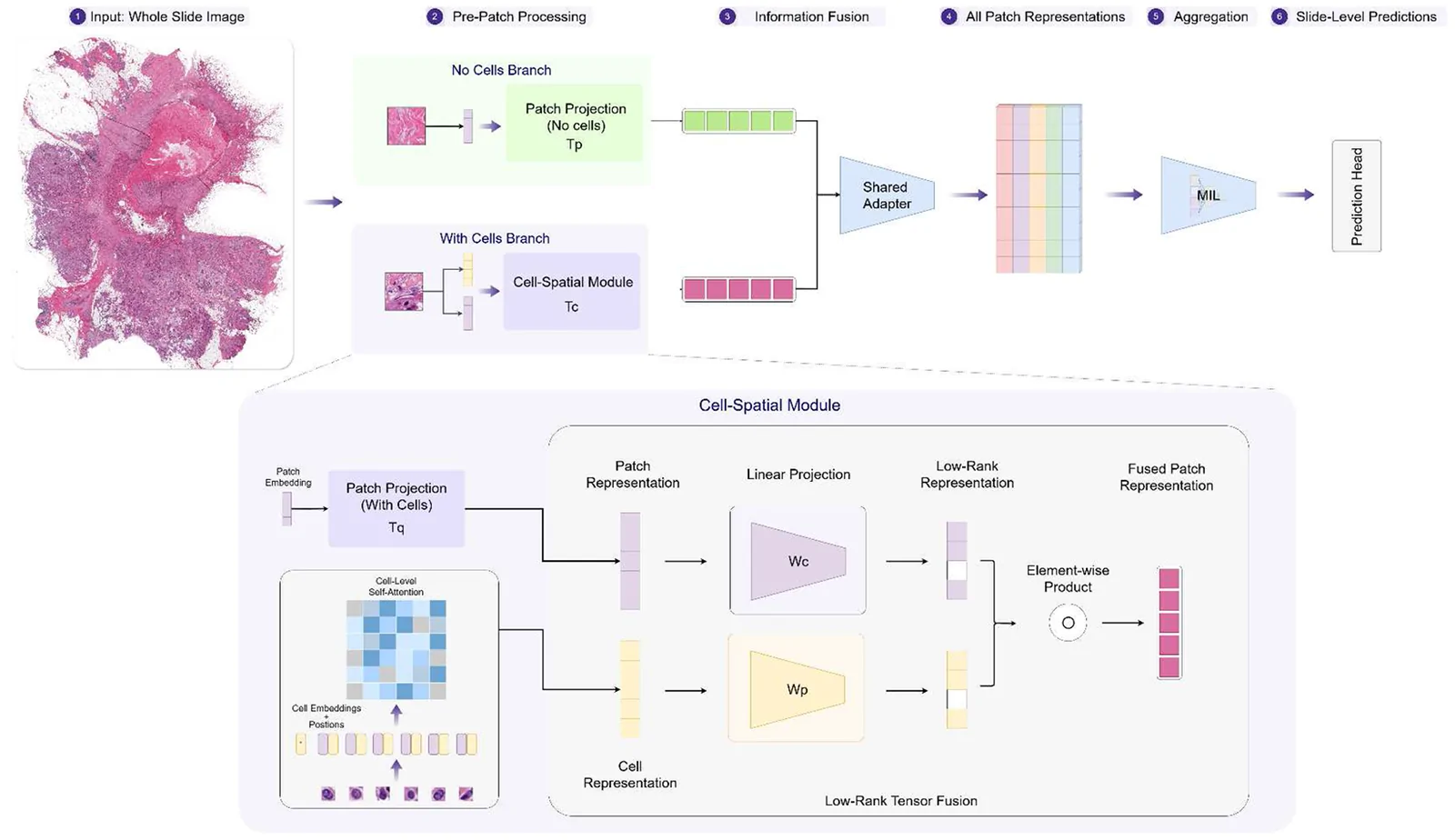
Architecture of the CellPlexer framework for biomarker-aware multimodal whole-slide image integration. **Overall CellPlexer multimodal workflow.** Whole-slide images are partitioned into fixed-size patches and processed through parallel morphology and cellular representation pathways. A pathology foundation model extracts high-dimensional tissue morphology embeddings, while CellViT simultaneously performs nuclear segmentation and generates per-cell embeddings alongside predicted biomarker phenotypes. Cell-level representations, spatial coordinates, and morphology embeddings are subsequently integrated through the CellPlexer fusion architecture before slide-level aggregation for downstream clinical prediction. **Spatial-aware cellular aggregation and biomarker integration module.** Individual cell embeddings are augmented using normalized spatial centroid coordinates encoded through sinusoidal positional embeddings to preserve local tissue topology. A transformer-based self-attention mechanism aggregates neighboring cellular interactions into a unified contextual representation summarizing the local tumor microenvironment. This representation is subsequently fused with the corresponding morphology-derived patch embedding, enabling simultaneous modeling of cellular composition, tissue organization, and histomorphological context. **Slide-level multimodal aggregation and interpretability.** Fused patch-level embeddings undergo low-rank tensor fusion followed by gated attention-based MIL aggregation to produce a global slide representation for patient-level prediction.

## Data Availability

The ORION dataset used for model training and internal validation is publicly available as described in the original publication, with image data and processing scripts accessible at https://zenodo.org/records/7637988. TCGA-BRCA whole-slide images and associated clinical data are publicly available through the National Cancer Institute Genomic Data Commons at https://portal.gdc.cancer.gov/. The public CRC-SurGen cohort is available through the SurGen dataset at https://github.com/CraigMyles/SurGen-Dataset. HEST-1k, used for independent transcriptome-based validation, is publicly available at https://huggingface.co/datasets/MahmoodLab/hest. The internal triple-negative breast cancer (TNBC) and invasive lung adenocarcinoma (ILA) cohorts contain patient-level clinical and imaging data and are maintained under institutional data-use agreements; they cannot be publicly redistributed by the authors but are available from the corresponding author upon reasonable request, subject to institutional approval and any applicable data-use restrictions. The numerical source data underlying the graphs and statistical analyses presented in this study are provided with the manuscript as Supplementary Data.

## Code Availability

The source code used to develop and evaluate CellPlexer is publicly available at https://github.com/AI4Path-Lab/CellPlexer. Additional implementation materials generated in this study are available from the corresponding author upon reasonable request, subject to any applicable institutional or data-use restrictions.
